# Ciprofloxacin-Induced Leukocytoclastic Vasculitis

**DOI:** 10.7759/cureus.78662

**Published:** 2025-02-07

**Authors:** André Cabrita, Pedro Lisboa Gonçalves, Catarina A Marques

**Affiliations:** 1 Cardiology Department, Unidade Local De Saúde São João, Porto, PRT; 2 Nephrology Department, Unidade Local De Saúde São João, Porto, PRT

**Keywords:** adverse drug reactions, ciprofloxacin, drug-induced vasculitis, fluoroquinolones, leukocytoclastic vasculitis

## Abstract

Ciprofloxacin is a widely used empiric antibiotic that may cause adverse reactions, including leukocytoclastic vasculitis (LCV). We present the case of a 51-year-old woman diagnosed with pyelonephritis, who developed cutaneous vasculitic lesions one day after ciprofloxacin administration. The differential diagnosis was broad and included autoimmune diseases, which were ruled out through extensive testing. A skin biopsy confirmed LCV. This rare drug-induced reaction was successfully managed by discontinuing ciprofloxacin, and the patient made a full recovery within five days without the need for corticosteroid treatment.

## Introduction

Ciprofloxacin, a second-generation fluoroquinolone, is widely utilized in clinical practice due to its efficacy in treating a range of bacterial infections, including urinary tract infections, respiratory infections, and gastrointestinal conditions. It exerts its antibacterial action by inhibiting bacterial DNA gyrase and topoisomerase IV, essential enzymes for DNA replication and transcription. Recent studies indicate a decline in the efficacy of ciprofloxacin against nosocomial urinary tract infections, possibly due to antimicrobial resistance, with low ciprofloxacin sensitivity rates against pathogens such as *Escherichia coli, Klebsiella pneumoniae*, and *Enterococcus faecium/faecalis*. Despite its broad therapeutic benefits, ciprofloxacin is not without risks. Its adverse effects are diverse, ranging from mild gastrointestinal disturbances to more severe complications like tendon rupture, peripheral neuropathy, and cardiac arrhythmias. The incidence of skin reactions is rare, occurring in approximately 1% of patients, and may include conditions such as erythema multiforme and, more infrequently, leukocytoclastic vasculitis (LCV) [1].

LCV, a small vessel vasculitis, is characterized by inflammation of the small blood vessels in the skin, leading to palpable purpura, petechiae, and other vascular lesions. The condition is often triggered by infections, autoimmune diseases, or, more rarely, medications [2]. Ciprofloxacin-induced LCV has been reported only sporadically in the literature, with most cases presenting as isolated skin reactions [3]. This article highlights a case of LCV induced by ciprofloxacin, demonstrating the challenges in diagnosis and management, and emphasizing the importance of drug withdrawal in the resolution of this rare side effect.

## Case presentation

A 51-year-old caucasian woman, with no known history of any illness or drug allergy, and no usual medication, presented with dysuria, lower back pain, and low-grade fever (37.9ºC). Initially misdiagnosed with an uncomplicated urinary tract infection, she was treated with 3000 mg of fosfomycin once daily for two days. As there was no symptomatic improvement, the patient returned to the basic health unit and was diagnosed with pyelonephritis. Ciprofloxacin (500 mg twice a day) was prescribed for five days.

**Figure 1:**
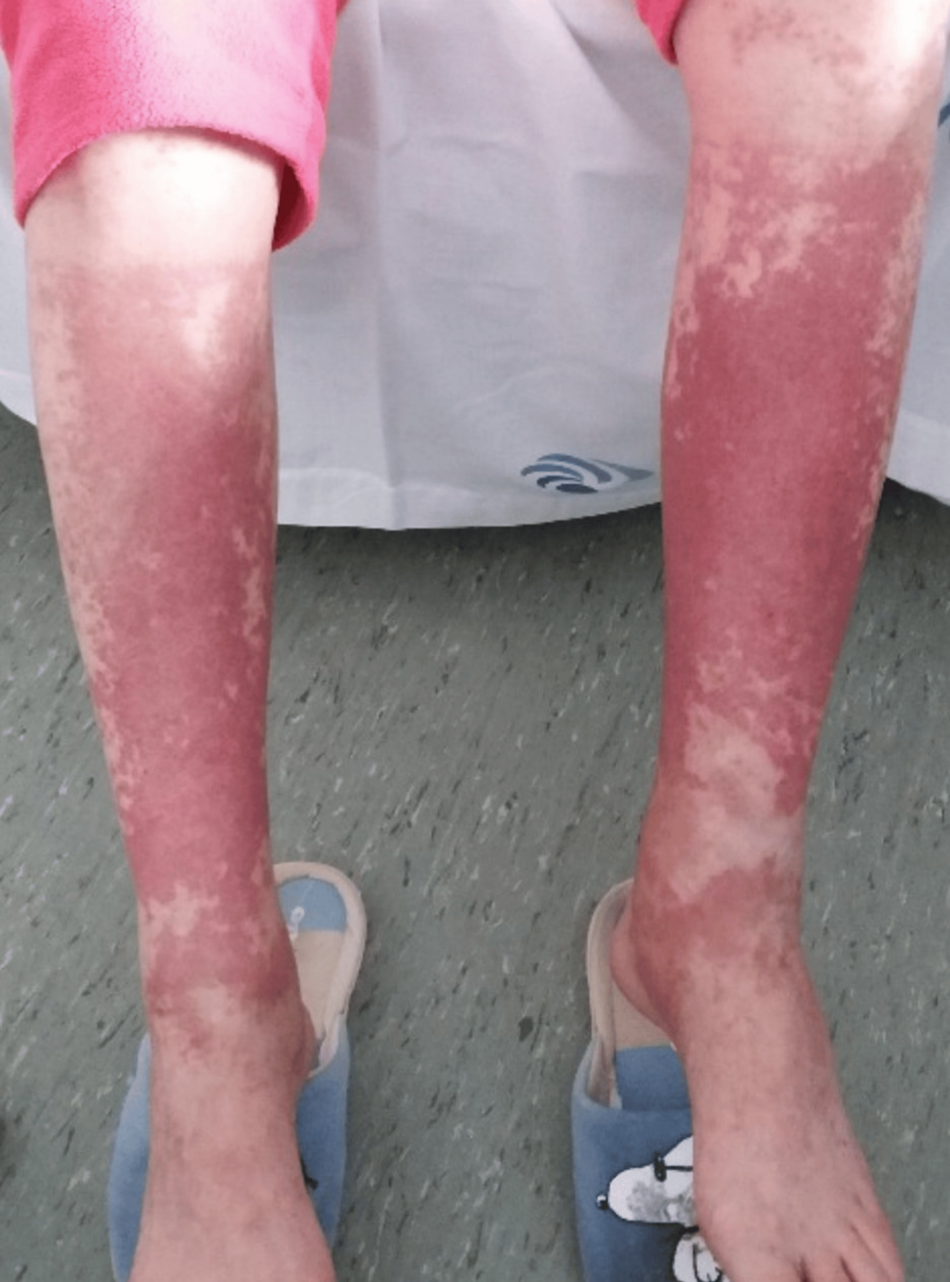
Severe rash on lower limbs upon presentation, one day after ciprofloxacin administration.

One day after the first dose of ciprofloxacin, the patient developed a non-pruritic rash, mostly on her lower legs and feet, which was initially small but rapidly expanded. Upon presentation to the emergency department (ED), her physical exam revealed non-blanching and tender lesions on her lower limbs (Figure 1).

Laboratory tests showed slight leukocytosis and elevated C-reactive protein, indicative of systemic inflammation (Table 1).

**Table 1:** Laboratory tests. Laboratory tests showed slight leukocytosis (13.08 × 10^9^/L) and elevated C-reactive protein (159.9 mg/L).

Substance	Measured value	Reference value
Leucocytes	13.08 × 10^9^/L	3.5-10^9^/L
C-reactive protein	159.9 mg/L	<3 mg/L

During hospitalization, a more comprehensive analytical study was carried out. Mycobacteriological tests of urine and blood were negative, as were serological tests. No change was detected in the value of immunoglobulins or autoimmune studies (namely antinuclear antibodies, anti-DsDNA, ANCA, anti-glomerular basement membrane, anti-mitochondrial, and anti-smooth muscle), with the exception of rheumatoid factor, which was slightly increased (270 IU/mL). The 24-hour urine study showed mild proteinuria (240 mg), with a normal protein-creatinine ratio. We interpreted this result as a consequence of urinary concentration and remaining signs of urinary tract infection. Since the patient never had renal dysfunction or other suggestive anomalies, we considered it unlikely that it was of autoimmune or glomerular origin. We performed a skin biopsy, the result of which was only available after the patient’s discharge, and demonstrated histological changes compatible with VLC.

**Figure 2:**
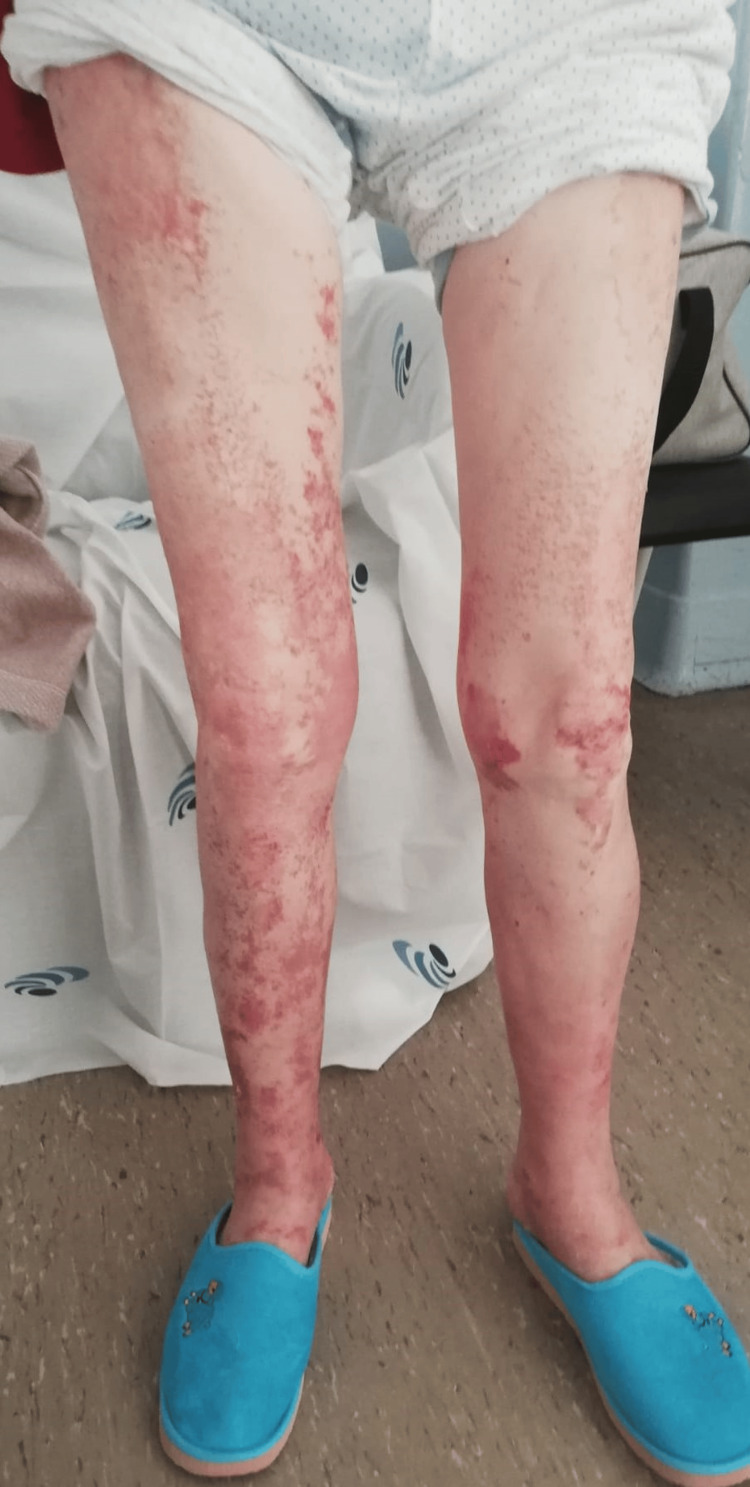
Significant improvement in the rash on the lower limbs by the fourth day after discontinuing ciprofloxacin.

**Figure 3:**
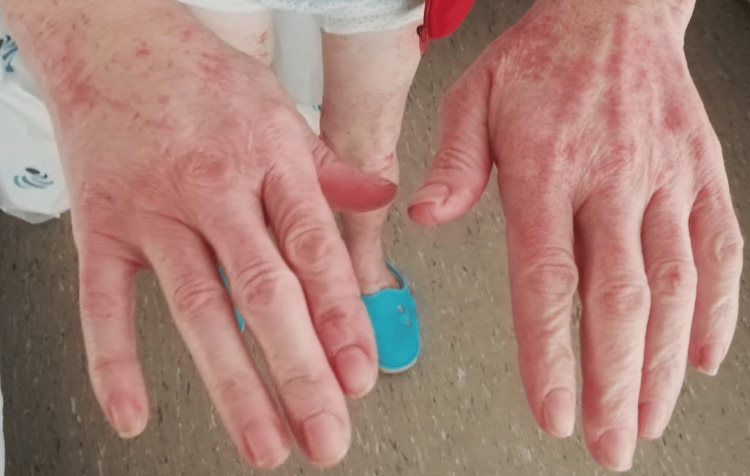
Minimal rash on the hands observed on the fourth day after discontinuing ciprofloxacin.

The treatment strategy was designed immediately after admission and before the results of the skin biopsy, given the temporal relationship between the administration of ciprofloxacin and the development of symptoms. Treatment consisted only of discontinuation of ciprofloxacin, without the administration of corticosteroids. The rash resolved spontaneously, with significant improvement on day 4 after stopping ciprofloxacin (Figure 2 and Figure 3).

After five days, the skin lesions completely disappeared, and the patient was discharged without subsequent complications.

## Discussion

Ciprofloxacin-induced LCV is an uncommon but important diagnosis to consider in patients who develop unexplained skin lesions during ciprofloxacin therapy. LCV induced by ciprofloxacin occurs due to a type III hypersensitivity mechanism, in which immune complexes are deposited in the walls of small blood vessels. Ciprofloxacin can act as a hapten or trigger an exaggerated immune response, leading to the formation of antigen-antibody complexes in circulation. These immune complexes tend to accumulate in the walls of small-caliber blood vessels, primarily in the skin, where they initiate an inflammatory response. Once deposited, these immune complexes activate the complement system, resulting in the release of C3a and C5a, which function as anaphylatoxins that recruit neutrophils to the affected site. The activated neutrophils then release proteolytic enzymes, reactive oxygen species, and pro-inflammatory cytokines, which contribute to endothelial injury, destruction of the vascular wall, and red blood cell extravasation. As a result, vascular damage leads to inflammation of the vessel walls, fibrinoid necrosis, and neutrophilic infiltration [4]. Most reported cases of ciprofloxacin-induced LCV present with purpuric lesions typically localized to the lower extremities, similar to the findings in our case [5].

The differential diagnosis for skin lesions in patients on ciprofloxacin is broad and includes conditions such as infectious vasculitis, autoimmune diseases, and other drug-induced reactions. In this case, we excluded conditions like systemic lupus erythematosus and vasculitis associated with other medications, as there were no supporting clinical or laboratory findings. The temporal relationship between ciprofloxacin administration and the onset of skin lesions strongly suggested a drug-induced etiology.

Management of drug-induced LCV typically involves discontinuing the offending medication. In some cases, corticosteroids may be indicated, particularly in more severe or systemic forms of vasculitis. However, corticosteroid therapy carries potential risks, including immunosuppression and other adverse effects. In this case, the patient responded promptly to ciprofloxacin withdrawal alone, allowing corticosteroids to be avoided. This approach is supported by reports that suggest drug withdrawal can result in the complete resolution of symptoms in non-critical cases [6].

In rare cases where systemic involvement is suspected, additional interventions such as corticosteroid therapy may be required, but this must be carefully weighed against the potential side effects of steroid use [7]. The Naranjo Adverse Drug Reaction Probability Scale, which assesses the likelihood of a drug causing an adverse reaction, was used in this case, yielding a score of seven, indicating a “probable” association between ciprofloxacin and the development of LCV.

## Conclusions

LCV is a rare but significant adverse reaction to ciprofloxacin. This case underscores the importance of considering ciprofloxacin in the differential diagnosis of vasculitis, particularly when a patient presents with skin lesions shortly after starting the medication. Drug discontinuation alone was sufficient to resolve the symptoms in this patient, highlighting the efficacy of this approach while avoiding the use of corticosteroids. Clinicians should be vigilant for potential drug-induced vasculitic reactions, and early identification and management are essential to prevent further complications.
